# Where Are You From?

**DOI:** 10.1007/s11606-025-10146-z

**Published:** 2026-01-20

**Authors:** Barat S. Venkataramany

**Affiliations:** Department of Internal Medicine, University of Cincinnati College of Medicine, Cincinnati, OH USA

The patient scheduled an appointment with me to discuss recent diarrhea and concerns for parasitic infection. I entered the exam room and met him for the first time, shaking his hand and introducing myself. Almost immediately, he asked: “Where are you from?” I answered truthfully: “Ohio.” I had never thought much of it and assumed people were genuinely curious, but some artful ways to probe at my ethnicity included “Where were you born?” or “What language does Venkataramany come from?” or “Where do your parents and grandparents live?” The patient followed up with “Where are your people from?” My people? I said that this was not relevant, but he persisted. “Of course it is…your people’s medicine is different.” Incredulous, I tried to direct his attention to the reason for his visit, but he continued to ask about where I obtained my medical degree, if I was actually a physician, and why Google knew more than me about his symptoms.

In 30 min, my identity, family, and whether I was even supposed to be in the clinic were questioned. As I returned to the workroom, stunned at what had transpired, the preceptors remarked that I looked as if I had seen a ghost. Quietly and shakily, I listed the incisive questions and comments. In the hopes of de-escalation, one of the preceptors and our nursing supervisor accompanied me when I returned to deliver the after-visit summary. When the patient was asked why he made comments about my ethnicity and qualifications, he adamantly denied saying anything of the sort. Minutes later, he pointed to me and told my preceptor, “It’s important to know where a doctor like him is from because I want to know that I am receiving the same standard of care as I would from a doctor like you.” Near tears, I left the room, followed by my preceptor. He recommended that I discuss this incident with clinic leadership. When I recounted the story to our clinic director the next day, he dismissed the patient from the clinic because he did not display the ability to treat all staff members with respect.

As I pondered “Where are you from?” and the charged questions that ensued, I realized that I had never seen an attending physician be asked those four words. I had to craft the answer on my own. At face value, the question seems innocuous. But as an Indian-American and part of the 52.8% of over 130,000 resident physicians who do not identify as White,^1^ this question is almost a normal part of the day. Unfortunately, it can devolve into foreigner objectification: the application of stereotypes to posit that ethnic minority individuals are foreigners.^2^ Despite being born and raised in the United States, I have encountered this several times, mostly through remarks of how I speak such good English without an accent. Asian- and Latinx-Americans are particularly susceptible to this phenomenon and such comments. The burden of these harmful experiences facilitates physician burnout, disillusionment, and moral injury.^3^

Despite these risks, we enter the workplace ready to uphold the principles of professionalism and conduct ourselves in manners befitting our roles. This is part of forming professional identity, defined as “a representation of self, achieved in stages over time during which the characteristics, values, and norms of the medical profession are internalized, resulting in an individual thinking, acting, and feeling like a physician.”^4^ This process is not without difficulties. Physicians who are underrepresented in medicine, particularly Black Americans, are tasked with forming professional identity within the challenges of sociohistorical contexts and systemic inequities.^5^ In forming this important aspect of self, trainees undertake sacrifice and undergo scrutiny through several formative years. We are often reminded that we should not lose ourselves to medicine, but we seem to neglect the importance of another continuous process that evolves with our development as physicians: personal identity. But who we are as people – individuals with emotions and experiences, not unlike the patients we treat – seems to take a back seat to the role of physician when we wear the badge that bears our credentials.

As we grapple with the challenge of shaping the interplay between these identities, we care for patients and place their needs above our own. In doing so, we recognize the role of implicit bias and the harm it can do to patient care. We are taught to always leave preconceived notions at the door. We are instructed to be cognizant of mental roadblocks as we care for individuals with markedly different experiences and values from ourselves. But when we face that same bias from patients – through subtleties or overtures against who we are – what are we to do? Physicians are not guaranteed space or time to reflect and process the harmful words that are leveled against them. But we do not acknowledge the toll that this can take, especially when such experiences accumulate. We are merely taught to counsel patients that these remarks are inappropriate, but this is insufficient. We need formal education to prepare trainees to respond to microaggressions or overt racism from patients instead of leaving them to struggle on their own in formulating the answers. This starts with honest conversations about microaggressions and bias against physicians and education on how to be proactive rather than passive.

I never received formal education on how to respond to a patient if a colleague or I were the victim of a macroaggression or microaggression. However, I did encounter emerging curricula on empowering trainees to respond to inappropriate patient behaviors and navigate the ethical dilemmas of these situations. Simple surveys to elicit experiences with witnessing and/or experiencing macroaggressions and microaggressions can promote speaking up.^6^ Instruments like the Discrimination 911 algorithm^7^ can help colleagues to be effective bystanders for those targeted by hurtful remarks in the workplace. While I would welcome programs to institute curricula such as these, the lesson from these tools is that large-scale organizational policies addressing inappropriate patient conduct are needed to protect physicians and their sense of mental well-being and belonging.^3^ I wonder if my colleagues or I would have intervened or responded differently had we had the opportunity to learn from trainings like those above. I wonder if even I would have responded differently had I undertaken formal education. I look forward to further educating myself and standing up the next time colleagues or I face macroaggressions and microaggressions.

While neither the medical profession nor I can eradicate the human curiosity to ask where someone is from, we should understand that these four words alone are not the problem. Rather, we should recognize that this question has the propensity to turn harmful and spiral into challenging a physician’s sense of personal and professional self, whether we are at risk of facing this question and its downstream effects or not. We should feel empowered to defend ourselves and our colleagues when we face bias and discrimination. Our profession must pursue education on these experiences and prepare and protect trainees in such moments. Not every patient encounter that sees this question will unfold like the one I faced, but if one were to, I hope that my colleagues and I will be well-equipped to respond with poise and professionalism.
